# Reported Use and Perceived Understanding of Sodium Information on US Nutrition Labels

**DOI:** 10.5888/pcd12.140522

**Published:** 2015-04-09

**Authors:** Jessica Lee Levings, Joyce Maalouf, Xin Tong, Mary E. Cogswell

**Affiliations:** Author Affiliations: Joyce Maalouf, Xin Tong, Mary E. Cogswell, Division for Heart Disease and Stroke Prevention, National Center for Chronic Disease Prevention and Health Promotion, Centers for Disease Control and Prevention, Atlanta, Georgia.

## Abstract

**Introduction:**

Comparing nutrition labels and choosing lower sodium foods are tactics to help reduce excessive sodium intake, a major risk factor for hypertension. Our objective was to assess US adult consumers’ reported use and perceived understanding of sodium information on nutrition labels by sociodemographic and health status.

**Methods:**

We analyzed responses to questions from 3,729 adults aged 18 years or older participating in 2 national cross-sectional mail panel surveys in 2010.

**Results:**

We found that 19.3% (95% confidence interval [CI], 17.2%–21.6%) of respondents agreed they were confused about how to figure out how much sodium is in the foods they eat; 57.9% (95% CI, 55.4%–60.5%) reported that they or the person who shops for their food buy items labeled low salt or low sodium; and 46.8% (95% CI, 44.3%–49.4%) reported they check nutrition labels for sodium content as a tactic to limit salt. Consumers with a high school education or less were more likely than college graduates to report they were confused about sodium content on labels (adjusted odds ratio [AOR], 1.9; 95% CI, 1.4–2.8) and less likely to check labels for sodium as a tactic to limit salt intake (AOR, 0.7; 95% CI, 0.6–0.98).

**Conclusion:**

Most survey respondents in our study reported buying low sodium food items. However, a higher proportion of respondents with low education than respondents with high education reported confusion with and less use of sodium content information, suggesting enhanced efforts may be needed to assist this group. Opportunity exists for health care professionals to educate patients about using and understanding nutrition labels and consuming a diet consistent with the Dietary Approaches to Stop Hypertension (DASH) eating plan.

## Introduction

People who report using sodium information on food labels consume significantly less sodium than those who do not use such information (1). In a 2012 Web-based survey conducted by the International Food Information Council (IFIC), 37% of US consumers reported regularly purchasing products labeled low sodium (2). Additionally, 2014 IFIC data indicated that 95% of US consumers believe sodium information on the Nutrition Facts label is very or somewhat helpful when making decisions about what foods to buy and that more people are trying to limit salt or sodium than are trying to limit calories, sugars, or fats (3). This survey and others suggest that 53% to 58% of consumers limit or are trying to limit, restrict, or avoid salt/sodium (3–5). In the United States, packaged and restaurant foods are the primary source of dietary sodium (6–8). Both the 2010 *Dietary Guidelines for Americans* and *Healthy People 2020* recommend reducing the average US sodium intake, which is currently well above recommended levels (9,10). Excess sodium intake can increase the risk of high blood pressure and subsequent cardiovascular diseases, the leading causes of death in the United States (11). In a 2010 report, the Institute of Medicine (IOM) recommended revising and updating nutrition labels and monitoring consumers’ “ability to estimate sodium intake,” and the US Food and Drug Administration is proposing to update the Nutrition Facts label found on most packaged food items in the United States; if adopted, one of the proposed changes would reduce the Daily Value for sodium from 2,400 mg to 2,300 mg. Data on consumer’s perceived understanding, confusion, and reported use of sodium information on nutrition labels for purchases can serve as a baseline for helping to evaluate the impact of proposed changes. To our knowledge, researchers have not evaluated consumers’ attitudes and beliefs about their understanding of sodium content on nutrition labels. We hypothesized that adults with a higher risk of heart disease would be more likely to report using sodium information on labels and that those with lower education would be less likely to report understanding sodium information on labels. The primary objective of this analysis was to describe US adult consumers’ self-reported use and perceived understanding of sodium information on nutrition labels (both on the Nutrition Facts label and on the front of food packages) by sociodemographic and health characteristics.

## Methods

With technical assistance from the Centers for Disease Control and Prevention (CDC), we submitted 5 questions to the 2010 ConsumerStyles and 2 questions to the HealthStyles cross-sectional mail panel surveys conducted by the public relations firm Porter Novelli. We linked data from both surveys to evaluate responses from the same participants to 6 statements and 1 question about self-reported confusion, knowledge, and use of nutrition labels to reduce sodium intake. The ConsumerStyles survey was conducted in April and May 2010. Participants were selected according to region of residence, annual household income, population density, age, and household size to create a diverse national sample. Of the 20,000 people selected, 10,328 (51.6%) responded. The HealthStyles survey was conducted in September and October 2010; 6,253 people who responded to the 2010 ConsumerStyles survey were randomly selected to participate, and 4,198 (67.1%) responded. Samples from the 2010 HealthStyles survey were weighted for age, sex, race/ethnicity, annual household income, and household size to represent the US Census Bureau’s estimated US population for 2009. For this study, data from the 2 surveys were merged and a subsample of people responding to the 2010 HealthStyles survey was used. Of the 4,198 HealthStyles respondents, we consecutively excluded the following: 237 (5.6%) respondents with incomplete responses on at least 1 of the survey statements or questions of interest, 25 respondents missing information on education, 120 respondents missing information on smoking status, 48 respondents missing information on height, and 39 respondents missing information on weight. This process yielded 3,729 respondents. Respondents included in our analyses did not differ from those excluded (n = 469) in terms of sex, education level, region of residence, body mass index (BMI), or diabetes diagnosis (Appendix). The possible responses to the question on smoking were the following: “former smoker,” “nonsmoker” and “smoker.” The categories “nonsmoker” and “former smoker” were combined.

A higher proportion of included respondents than excluded respondents were aged 18 to 50 (60.5% vs 48.8%; *P* < .001), were white non-Hispanic (69.8% vs 58.9%; *P* = .02), had an annual household income of $60,000 or more (44.2% vs 29.9% ; *P* < .001), and were nonsmokers (84.1% vs 77.3%; *P* = .055 ); a lower proportion of included respondents reported high blood pressure (28.2% vs 35.7%; *P* = .02) (Appendix). 

This study was deemed exempt from institutional review board approval under federal regulation 45 CFR §46.101(b).

During the ConsumerStyles survey, participants were asked to use a 5-point Likert scale to agree or disagree with the following 4 statements: “I specifically buy foods labeled low or reduced salt/sodium,” “I am confused about how to figure out how much sodium is in the food I eat,” “Information on nutrition labels often helps me decide what food to buy,” and “I am confused about how to use the Nutrition Facts label to figure out how much sodium is in the food I eat.” Because the objective of our study focused on sodium information, we did not analyze data on the third question (whether nutrition labels helped participants to decide in general what food to buy). Participants also were asked to answer yes or no to the following statement: “I check nutrition labels for sodium content as a tactic to lower the salt in my diet.” During the HealthStyles survey, participants were asked to use a 5-point Likert scale to agree or disagree with the following statement: “I know how to monitor the amount of salt I eat based on the information provided on nutrition labels.” They were also asked to respond yes or no to the following question: “Do you or the person who shops for your food buy items that are labeled ‘low salt’ or ‘low sodium’?”

The surveys also included questions about respondents’ sociodemographic and health characteristics. Self-reported sociodemographic characteristics included (but were not limited to) age, sex, race/ethnicity, annual household income, education level, and region of residence. Self-reported health characteristics included height, weight, smoking status, diagnosed diabetes, and diagnosed high blood pressure. Height and weight were used to calculate each respondent’s body mass index ([BMI], weight in kg/height in m^2^).

Weighted percentages and 95% confidence intervals (CIs) were calculated for the responses to the survey questions by sociodemographic and health characteristics. We used χ^2^ tests to assess differences in responses between subgroups, where an α level of .05 was considered significant. Multiple logistic regression analyses were conducted for each question to determine the associations with sociodemographic and health characteristics. Adjusted odds ratios (AORs) and 95% CIs were obtained from each model after controlling for age, sex, race/ethnicity, annual household income, education level, region of residence, BMI, smoking status, diagnosed diabetes, and diagnosed high blood pressure. Preliminary analyses of the 5-point Likert responses were conducted to examine the AORs for “agree” versus the 2 other responses (“neither agree nor disagree” and “disagree”). Responses of “strongly agree,” “moderately agree,” and “somewhat agree” were combined into 1 “agree” category. All statistical analyses were performed using the following statistical software: SPSS Statistics (version PASW18, 2010; IBM Corp) and SAS (version 9.2, 2012; SAS Institute Inc). SPSS was used to run the descriptive analyses and χ^2^ tests, and SAS was used to generate logistic regressions and to replicate the main results.

## Results

Reported understanding of how to monitor sodium content in foods varied by question (Table 1). About 1 in 5 (19.3%) respondents agreed they were confused about how to figure out how much sodium is in the food they eat (29% neither agreed nor disagreed, and 52% disagreed), and 1 in 8 (13.2%) respondents agreed that they were confused about how to use Nutrition Facts label to figure out how much sodium is in the food they eat (23% neither agreed nor disagreed, and 64% disagreed) (Table 1). Most (55.8%) agreed they knew how to monitor the amount of salt they eat based on the information provided on nutrition labels (28% neither agreed nor disagreed, and 16% disagreed). After adjusting for other characteristics, respondents with a high school education or less were more likely than college graduates to agree they were confused about how to figure out how much sodium is in their food (Table 1), as were adults aged 71 or older (compared with adults aged 18 to 50), black non-Hispanics (compared with white non-Hispanics), those with an annual household of income $15,000 or less (compared with those with an income of ≥$60,000), and those with a diabetes diagnosis (compared with those who did not report having diabetes). Similarly, when asked about confusion with how to use nutrition facts labels to figure out sodium content, respondents aged 51 or older (compared with respondents aged 18–50); black non-Hispanics, Hispanics, and those of “other” race/ethnicity (compared with white non-Hispanics), those with an annual household income of less than $15,000 (compared with those with an income of ≥$60,000), and non-college graduates (compared with college graduates) were more likely to agree they were confused. Women were more likely than men to report knowing how to monitor the amount of salt they eat based on nutrition labels but were not less likely to agree they were confused.

**Table 1:** Confusion About and Knowledge of Sodium Information on Food Labels Among Selected Respondents (n = 3,729) to ConsumerStyles and HealthStyles Surveys, 2010^a^

Characteristic	I am confused about how to figure out how much sodium is in the food I eat.		I am confused about how to use Nutrition Facts label to figure out how much sodium is in the food I eat.		I know how to monitor the amount of salt I eat based on the information provided on nutrition labels.	
	Agree, % (95% CI)^b^	AOR (95% CI)	Agree, % (95% CI)^b^	AOR (95% CI)	Agree, %(95% CI)^b^	AOR (95% CI)
**Overall**	19.3 (17.2–21.6)	—	13.2 (11.8–14.8)	—	55.8 (53.2–58.4)	—
**Age, y**						
18–50	18.0 (14.8–21.6)^c^	1.0 [Reference]	10.8 (8.8–13.1)^d^	1.0 [Reference]	53.8 (49.8–57.7)^c^	1.0 [Reference]
51–70	19.0 (16.9–21.2)	1.05 (0.8–1.3)	16.0 (14.0–18.1)	1.7 (1.3–2.3)	60.5 (57.7–63.2)	1.2 (1.0–1.4)
≥71	28.8 (24.7–33.3)	1.9 (1.4–2.6)	20.0 (16.4–24.1)	2.3 (1.6–3.2)	54.5 (49.7–59.1)	0.9 (0.7–1.2)
**Sex**						
Male	19.1 (16.7–21.7)	1.0 [Reference]	12.7 (10.9–14.8)	1.0 [Reference]	51.7 (48.0–55.4)^e^	1.0 [Reference]
Female	19.5 (16.3–23.2)	1.05 (0.8–1.4)	13.7 (11.6–16.1)	1.1 (0.8–1.4)	59.7 (56.0–63.3)	1.5 (1.2–1.8)
**Race/ethnicity**						
White, non-Hispanic	16.5 (14.2–19.0)^d^	1.0 [Reference]	11.5 (9.9–13.3)^e^	1.0 [Reference]	58.1 (55.0–61.2)	1.0 [Reference]
Black, non-Hispanic	31.4 (22.9–41.4)	2.1 (1.3–3.5)	17.5 (13.2–22.8)	1.5 (1.01–2.1)	50.4 (41.3–59.4)	0.7 (0.5–1.1)
Hispanic	22.0 (17.5–27.3)	1.4 (0.97–2.0)	16.0 (12.3–20.6)	1.4 (1.01–2.1)	50.4 (43.9–57.0)	0.8 (0.6–1.04)
Other^f^	23.9 (17.1–32.3)	1.6 (1.02–2.4)	19.6 (13.3–28.0)	1.8 (1.01–3.3)	51.3 (41.7–60.8)	0.8 (0.5–1.1)
**Annual household income, $**						
<15,000	35.2 (29.6–41.3)^d^	1.9 (1.3–2.8)	23.6 (18.8–29.2)^d^	1.8 (1.2–2.7)	53.4 (47.2–59.5)^c^	0.9 (0.6–1.2)
15,000–24,900	20.4 (14.5–27.9)	0.9 (0.6–1.4)	20.0 (14.5–26.8)	1.5 (0.9–2.5)	61.4 (51.6–70.3)	1.2 (0.8–1.9)
25,000–39,900	20.5 (14.2–28.7)	1.06 (0.7–1.4)	13.2 (10.1–17.1)	1.1 (0.7–1.6)	46.9 (40.0–53.9)	0.7 (0.5–0.9)
40,000–59,900	16.4 (11.0–23.6)	0.9 (0.6–1.4)	9.9 (7.6–12.9)	0.8 (0.6–1.2)	54.0 (47.9–60.0)	0.8 (0.6–1.1)
≥60,000	15.5 (13.2–18.1)	1.0 [Reference]	10.0 (8.1–12.3)	1.0 [Reference]	59.0 (55.3–62.7)	1.0 [Reference]
**Education level**						
≤High school graduate	25.6 (21.0–30.9)^d^	1.7 (1.2–2.5)	18.5 (15.4–22.1)^d^	1.9 (1.4–2.8)	54.0 (48.9–59.0)^c^	0.8 (0.6–1.01)
Some college	19.7 (16.3–23.7)	1.4 (0.99–1.9)	14.0 (11.5–17.0)	1.6 (1.1–2.3)	53.1 (48.5–57.7)	0.7 (0.6–0.95)
College graduate	13.9 (11.8–16.4)	1.0 [Reference]	8.2 (6.7–10.0)	1.0 [Reference]	60.5 (56.8–64.2)	1.0 [Reference]
**Region**						
Northeast	18.1 (14.0–23.1)	0.98 (0.6–1.5)	14.5 (11.3–18.5)	1.1 (0.8–1.7)	56.4 (50.0–62.7)	0.9 (0.7–1.3)
Midwest	19.1 (14.7–24.5)	0.99 (0.7–1.5)	11.0 (8.7–13.7)	0.8 (0.6–1.1)	53.2 (48.2–58.1)	0.9 (0.7–1.1)
South	20.8 (17.3–24.8)	1.0 [Reference]	14.2 (11.8–17.1)	1.0 [Reference]	56.8 (52.2–61.3)	1.0 [Reference]
West	17.6 (14.4–21.4)	0.9 (0.6–1.3)	12.8 (9.9–16.4)	0.9 (0.6–1.3)	56.7 (51.9–61.5)	1.0 (0.8–1.3)
**Body mass index, kg/m^2^**						
<25.00	16.8 (13.2–21.3)^c^	1.0 [Reference]	12.1 (9.5–15.2)^c^	1.0 [Reference]	56.7 (51.7–61.6)	1.0 [Reference]
25.00–29.99	17.8 (15.0–21.0)	1.1 (0.7–1.5)	11.1 (9.0–13.5)	0.9 (0.6–1.3)	54.9 (50.3–59.3)	1.0 (0.8–1.3)
≥30.00	23.0 (19.3–27.2)	1.3 (0.9–1.9)	16.3 (13.9–19.1)	1.3 (0.9–1.8)	56.0 (51.8–60.0)	0.9 (0.7–1.3)
**Current smoking status^g^**						
No	19.0 (16.7–21.5)	1.0 [Reference]	12.6 (11.2–14.2)	1.0 [Reference]	56.2 (53.3–59.0)	1.0 [Reference]
Yes	21.0 (16.3–26.6)	1.1 (0.7–1.6)	16.5 (12.2–21.9)	1.3 (0.8–1.9)	54.2 (48.1–60.2)	0.98 (0.7–1.3)
**Diagnosed diabetes**						
No	17.9 (15.7–20.4)^d^	1.0 [Reference]	12.3 (10.8–14.0)^d^	1.0 [Reference]	55.3 (52.4–58.2)	1.0 [Reference]
Yes	29.7 (25.1–34.7)	1.5 (1.1–2.0)	20.1 (16.6–24.1)	1.2 (0.9–1.7)	59.7 (54.7–64.5)	1.2 (0.9–1.6)
**Diagnosed high blood pressure**						
No	17.6 (15.0–20.7)^c^	1.0 [Reference]	11.9 (10.2–13.9)^e^	1.0 [Reference]	55.0 (51.6–58.4)	1.0 [Reference]
Yes	23.5 (20.9–26.3)	1.0 (0.8–1.3)	16.5 (14.3–18.9)	0.9 (0.7–1.2)	58.0 (54.7–61.3)	1.1 (0.9–1.4)

Abbreviations: AOR, adjusted odds ratio; CI, confidence interval.

a All estimates are weighted for age, sex, race/ethnicity, annual household income, and household size. The model included age, sex, race/ethnicity, annual household income, education level, region of residence, body mass index, smoking status, diagnosis of diabetes, and diagnosis of high blood pressure.

b Responses were offered on a 5-point Likert scale of “strongly agree,” “moderately agree,” “somewhat agree,” “neither agree nor disagree” and “disagree.” “Strongly agree,” “moderately agree,” and “somewhat agree” were combined into one “agree” category.

c χ^2^ test *P* < .05.

d χ^2^ test *P* < .001.

e χ^2^ test *P* < .01.

f Other race includes Alaska Native, American Indian, Asian, Native Hawaiian, and Other Pacific Islander.

g The possible responses to the question on smoking were the following: “former smoker,” “nonsmoker”, and “smoker.” The categories “nonsmoker” and “former smoker” were combined.

Reported use of sodium information on nutrition labels also varied by question (Table 2). About 1 in 3 (35.6%) respondents agreed they specifically buy foods labeled low or reduced salt or sodium (28% neither agreed nor disagreed, and 37% disagreed), and a little less than half (46.8%) said they check nutrition labels for sodium content as a tactic to lower salt in their diet. Most (57.9%) said they or the person who shops for their food buy items that are labeled low salt or low sodium.

**Table 2:** Consumer Use of Food Labels to Monitor Dietary Sodium Intake Among Selected Respondents (n = 3,729) to ConsumerStyles and HealthStyles Surveys, 2010^a^

Characteristic	I specifically buy foods labeled low or reduced salt/sodium.		I check nutrition labels for sodium as a tactic to lower salt in my diet.		Do you or the person who shops for your food buy items that are labeled “low salt” or “low sodium.”	
	Agree, %(95% CI)^b^	AOR (95% CI)	Yes, % (95% CI)^c^	AOR (95% CI)	Yes, % (95% CI)^c^	AOR (95% CI)
**Overall**	35.6 (33.1–38.1)	—	46.8 (44.3–49.4)	—	57.9 (55.4–60.5)	—
**Age, y**						
18–50	30.3 (26.6–34.3)^d^	1.0 [Reference]	43.3 (39.4–47.3)^e^	1.0 [Reference]	52.4 (48.4–56.3)^e^	1.0 [Reference]
51–70	40.8 (38.1–43.6)	1.4 (1.1–1.7)	50.3 (47.5–53.1)	1.2 (1.02–1.5)	64.7 (62.0–67.3)	1.4 (1.2–1.8)
≥71	52.6 (47.9–57.3)	2.2 (1.7–3.0)	58.1 (53.4–62.7)	1.7 (1.3–2.3)	72.0 (67.6–76.1)	2.0 (1.5–2.7)
**Sex**						
Male	33.6 (30.5–37.0)	1.0 [Reference]	43.3 (39.8–7.0)^d^	1.0 [Reference]	56.7 (53.0–60.3)	1.0 [Reference]
Female	37.4 (33.8–41.2)	1.2 (1.01–1.6)	50.0 (46.4–53.6)	1.4 (1.1–1.7)	59.1 (55.6–62.6)	1.1 (0.9–1.4)
**Race/ethnicity**						
White non-Hispanic	33.2 (30.4–36.1)^d^	1.0 [Reference]	46.6 (43.5–49.6)	1.0 [Reference]	56.1 (53.1–59.1)^e^	1.0 [Reference]
Black non-Hispanic	46.2 (37.2–55.4)	1.7 (1.1–2.5)	48.7 (39.7–57.7)	1.1 (0.8–1.5)	72.2 (65.5–78.0)	2.1 (1.4–3.1)
Hispanic	35.8 (30.0–42.1)	1.2 (0.9–1.7)	45.3 (38.8–52.0)	0.9 (0.7–1.3)	60.1 (53.3–66.6)	1.2 (0.9–1.7)
Other^f^	43.5 (34.0–53.4)	2.0 (1.2–3.1)	49.7 (40.2–59.3)	1.3 (0.9–2.0)	47.8 (38.6–57.2)	0.8 (0.6–1.2)
**Annual household income, $**						
<15,000	37.9 (32.3–43.9)	1.1 (0.8–1.5)	48.9 (42.8–55.1)^g^	1.2 (0.8–1.6)	57.3 (51.1–63.3)	0.8 (0.6–1.2)
15,000–24,900	31.5 (24.3–39.7)	0.8 (0.5–1.2)	35.2 (28.0–43.1)	0.6 (0.4–0.9)	55.2 (45.6–64.4)	0.8 (0.7–1.4)
25,000–39,900	41.4 (34.3–49.0)	1.4 (0.97–1.9)	53.0 (45.9–59.9)	1.3 (0.97–1.8)	60.2 (53.2–66.8)	1.04 (0.7–1.4)
40,000–59,900	38.7 (32.7–45.1)	1.3 (0.9–1.8)	48.1 (42.1–54.1)	1.1 (0.8–1.5)	59.1 (53.2–64.6)	1.05 (0.8–1.4)
≥60,000	32.7 (29.4–36.2)	1.0 [Reference]	46.5 (42.9–50.2)	1.0 [Reference]	57.6 (53.9–61.2)	1.0 [Reference]
**Education level**						
≤High school graduate	35.1 (30.2–40.3)	0.9 (0.7–1.2)	40.8 (35.9–45.9)^g^	0.7 (0.6–0.98)	55.8 (50.7–60.8)	0.9 (0.7–1.2)
Some college	36.3 (32.0–40.9)	1.1 (0.8–1.4)	49.9 (45.3–54.4)	1.1 (0.9–1.4)	58.6 (54.2–62.9)	1.00 (0.8–1.3)
College graduate	35.1 (31.9–38.4)	1.0 [Reference]	47.7 (44.2–51.3)	1.0 [Reference]	58.8 (55.0–62.5)	1.0 [Reference]
**Region**						
Northeast	36.2 (30.7–42.0)	1.1 (0.8–1.4)	48.5 (42.3–54.7)	1.1 (0.8–1.5)	62.8 (56.6–68.6^g^	1.2 (0.9–1.7)
Midwest	34.2 (29.5–39.4)	1.0 (0.7–1.3)	44.5 (39.6–49.5)	0.9 (0.7–1.2)	51.2 (46.3–56.1)	0.8 (0.6–1.00)
South	36.6 (32.4–41.1)	1.0 [Reference]	46.6 (42.2–51.1)	1.0 [Reference]	59.2 (54.7–63.6)	1.0 [Reference]
West	34.6 (30.1–39.4)	0.9 (0.7–1.2)	48.7 (43.9–53.6)	1.04 (0.8–1.4)	59.3 (54.4–64.0)	1.1 (0.8–1.4)
**Body mass index, kg/m^2^**						
<25.00	34.7 (30.2–39.5)	1.0 [Reference]	47.2 (42.4–52.0)	1.0 [Reference]	54.3 (49.5–59.1)	1.0 [Reference]
25.00–29.99	34.8 (31.2–38.6)	0.9 (0.7–1.2)	46.6 (42.2–51.0)	0.9 (0.7–1.2)	57.5 (53.0–61.9)	1.03 (0.8, 1.4)
≥30.00	37.2 (32.9–41.7)	0.9 (0.7–1.3)	46.7 (42.6–50.8)	0.9 (0.7–1.2)	61.8 (57.9–65.5)	1.2 (0.9–1.5)
**Current smoking status^h^**						
No	37.2 (34.5–40.0)^d^	1.0 [Reference]	48.1 (45.2–50.9)^g^	1.0 [Reference]	59.5 (56.7–62.3)^d^	1.0 [Reference]
Yes	27.0 (21.7–33.1)	0.7 (0.5–0.9)	40.3 (34.5–46.4)	0.8 (0.6–1.02)	49.6 (43.5–55.6)	0.8 (0.6–1.02)
**Diagnosed diabetes**						
No	34.1 (31.4–36.9)^e^	1.0 [Reference]	45.7 (42.9–8.5)^d^	1.0 [Reference]	56.0 (53.2–58.8)^e^	1.0 [Reference]
Yes	47.1 (42.0–52.2)	1.2 (0.96–1.6)	55.3 (50.1–60.3)	1.3 (1.05–1.7)	72.4 (67.4–76.9)	1.5 (1.2–2.0)
**Diagnosed high blood pressure**						
No	31.2 (28.1–34.4)^e^	1.0 [Reference]	44.7 (41.4–8.1)^d^	1.0 [Reference]	53.8 (50.5–57.1)^e^	1.0 [Reference]
Yes	46.8 (43.5–50.2)	1.6 (1.3–2.0)	52.1 (48.8–55.5)	1.2 (0.98–1.5)	68.4 (65.3–71.4)	1.4 (1.1–1.7)

Abbreviations: AOR, adjusted odds ratio; CI, confidence interval.

a Estimate percentages are weighted for age, sex, race/ethnicity, annual household income, and household size. The model included age, sex, race/ethnicity, annual household income, education level, region of residence, body mass index, smoking status, diagnosis of diabetes, and diagnosis of high blood pressure.

b Responses were offered on a 5-point Likert scale of “strongly agree,” “moderately agree,” “somewhat agree,” “neither agree nor disagree” and “disagree.” “Strongly agree,” “moderately agree,” and “somewhat agree” were combined into 1 “agree” category.

c Questions were answered as yes or no.

d χ^2^ test *P* < .01.

e χ^2^ test *P* < .001.

f Other race includes Alaska Native, American Indian, Asian, Native Hawaiian, and Other Pacific Islander.

g χ^2^ test *P* < .05.

h The possible responses to the question on smoking were the following: “former smoker,” “nonsmoker,” and “smoker.” The categories “nonsmoker” and “former smoker” were combined.

Reported use of sodium labeling typically found on the front of food packages (eg, “low sodium”) during shopping varied by sociodemographic and health characteristics (Table 2). The percentage of respondents who agreed that they specifically buy foods labeled low or reduced salt/sodium ranged from 27.0% (current smokers) to 52.6% (those aged ≥71). After adjusting for other characteristics, the likelihood of specifically buying foods labeled low or reduced salt/sodium was higher among respondents aged 51 or older than among those aged 18 to 50. The proportions agreeing were also higher among non-Hispanic blacks and those from “other” race/ethnicity than among non-Hispanic whites and among respondents who reported having high blood pressure than among those who did not report having high blood pressure.

About 7 of 10 respondents who were aged 71 or older, were non-Hispanic black, or who reported having diabetes or high blood pressure indicated they or the person who shops for their food buys items labeled low salt or low sodium (Table 2). After adjusting for other characteristics, the likelihood of reporting they or the person who shops for their food buy low-salt or low sodium items was higher among those aged 51 or older than among those aged 18 to 50, among non-Hispanic blacks than among non-Hispanic whites, and among those who reported having diabetes or high blood pressure than among those who did not report having those conditions.

## Discussion

This study suggests that less than 20% of US adult consumers are confused about how to figure out how much sodium is in the foods they eat and that more than half believe they know how to use nutrition labels to monitor the amount of salt they eat. Although these data are from 2010 they are useful in understanding consumers’ reported use and perceived understanding about the sodium content on nutrition labels. Consumer knowledge and understanding is unlikely to have changed between 2010 and 2015, given that no major education campaigns have taken place in the United States during this time and that our results on reported use of nutrition labels are consistent with the results of other studies (3–5).

Of some concern is that adults with less education or at higher risk of hypertension, such as older adults, non-Hispanic blacks, and those with diabetes, were more likely to be confused about how to figure out how much sodium is in the food they eat. Although most respondents to the 2010 ConsumerStyles and HealthStyles surveys reported that information on nutrition labels helps them decide what foods to buy (data not reported), results also suggest that most adults do not check Nutrition Facts labels as a tactic to lower salt in their diet. The difference in reported understanding and behavior could be related to a misunderstanding of 1 or both questions, use of nutrition labels to help make purchasing decisions not related to sodium, use of another tactic or strategy as the primary means to reduce sodium intake (eg, not adding salt at the table), or a lack of translation from knowledge to behavior because of other purchase considerations, such as time, preference, or cost. Not adding salt at the table is less effective than checking nutrition labels and choosing the lower-sodium option, because most sodium consumed in the United States is from sodium in packaged and restaurant foods, and only a small percentage is from salt added by the consumer. As hypothesized, respondents with less education were consistently more likely to agree they were confused about using Nutrition Facts labels to monitor their sodium intake, and less likely to check nutrition labels as a tactic to lower salt in their diet. These data extend findings from previous studies on the association of education with general use of food labels (12–14).

Our results suggesting that most US consumers or the person who shops for their food buy items labeled low salt or low sodium is consistent with other research (5,15) and suggests a demand for lower-sodium food choices (4) and the presentation of information on the front of the package to make choices. However, we do not know whether consumers are buying only 1 low sodium product or multiple low sodium products. A standardized front-of-package labeling system, similar to that proposed by the IOM in 2011, could help consumers make more healthful choices about their food purchases (16). Counseling consumers about reading and understanding food labels might be especially beneficial among populations with low socioeconomic status and among those who have risk factors for high blood pressure. Consumer knowledge of sodium and corresponding behavior change may be further influenced by counseling on the major sources of sodium and the Dietary Approaches to Stop Hypertension (DASH) eating plan. This approach may be especially beneficial among those who report having high blood pressure, who are already more likely to report that they or the person who shops for their food buy foods labeled low or reduced salt or sodium. However, because individual behavior change is difficult and because sodium is added to the food supply before foods are purchased, gradual reductions of sodium content by the food industry as a primary strategy recommended by the IOM to reduce US sodium intake would require little change on the part of the consumer.

Our study has several limitations. First, because ConsumerStyles and HealthStyles are mail panel surveys, they reach a population in which racial/ethnic minority and low-income households may be underrepresented. These surveys are based on a convenience sample of people willing to participate in a panel survey, and the characteristics of respondents to the survey or to certain questions may differ from the characteristics of the general population. Although the data were weighted to have the same distribution of key demographic characteristics as the distribution in the United States, they are not nationally representative. Even so, a previous study suggested that reported behaviors correlate well with representative and population-based surveillance data from CDC’s Behavioral Risk Factor Surveillance System (17). Second, because these surveys require literacy in English, people who do not speak English cannot participate. Third, respondents self-report their ability to understand nutrition labels, and the questions asked do not test the respondents’ actual knowledge. In addition, because the results of this study are based on self-reported data, they do not necessarily translate into consumer action. We do not know how the consumer uses information on sodium content to estimate their sodium intake. The strengths of this research include the large sample size, the contribution to new findings on consumers’ perceived understanding of sodium information on nutrition labels, and support of recommendations in a 2010 IOM report to strengthen and expand activities to measure population knowledge, attitudes, and behaviors about sodium among US consumers (7).

The results of this study suggest that most household food purchasers buy food items labeled low salt or low sodium, but fewer people — including members of subpopulations at high risk for high blood pressure and those with a high school education or less — check nutrition labels for sodium content as a tactic to limit sodium intake, and some adults are confused about how to determine the amount of sodium in foods. Food manufacturers can meet this demand by producing food items that are lower in sodium and including this information on the front of their packages. Doing so will offer greater choice and availability for the majority of consumers who want to buy low sodium products. Registered dietitians, health care professionals, and public health professionals can help by educating their clients and patients about the major sources of sodium in our diets, the importance of using nutrition labels to choose low-sodium foods, and how to understand and use nutrition labels.

## Appendix Comparison of Study Participants Whose Data Were Included in Analyses (n = 3,729) and Study Participants Who Were Excluded (n = 469), HealthStyles 2010

**Table:**

Characteristic	Included (n = 3,729)^a^		Excluded (n = 469)		χ^2^ *P* Value
	N^b^	%^c^ (95% CI)	N^b^	%^c^ (95% CI)	
**Age, y**					
18–50	1,752	60.5 (58.3–62.7)	163	48.8 (42.2–55.5)	<.001
51–70	1,478	29.9 (28.1–31.8)	199	34.0 (28.8–39.5)	
≥71	499	9.6 (8.7–10.6)	107	17.2 (13.8–21.2)	
**Sex**					
Male	1,825	48.0 (45.5–50.6)	232	47.4 (40.9–53.9)	.20
Female	1,904	52.0 (49.4–54.5)	237	52.6 (46.1–59.1)	
**Race/ethnicity**					
White non-Hispanic	2,590	69.8 (67.4–72.2)	281	58.9 (52.1–65.3)	.02
Black non-Hispanic	389	11.2 (9.4–13.2)	69	15.2 (11.0–20.7)	
Hispanic	405	13.1 (11.6–14.9)	69	17.7 (12.8–24.1)	
Other^d^	345	5.9 (4.8–7.1)	50	8.2 (5.2–12.7)	
**Annual household income, $**					
<15,000	518	11.7 (10.4–13.2)	114	23.0 (18.2–28.7)	<.001
15,000–24,900	300	11.3 (9.4–13.4)	59	15.6 (10.8–22.0)	
25,000–39,900	440	15.9 (14.0–18.1)	60	15.7 (11.2–21.6)	
40,000–59,900	591	16.8 (15.1–18.8)	64	15.8 (10.9–22.4)	
≥60,000	1,880	44.2 (41.7–46.8)	172	29.9 (25.0–35.2)	
**Education level**					
≤High school graduate	976	25.8 (23.6–28.1)	150	29.9 (24.8–35.5)	.32
Some college	1,386	40.5 (37.9–43.1)	154	40.0 (33.4–47.0)	
College graduate	1,367	33.8 (31.6–36.1)	137	30.1 (25.0–35.8)	
**Region**					
Northeast	684	18.5 (16.5–20.6)	76	13.7 (10.4–17.8)	.06
Midwest	901	24.5 (22.4–26.7)	98	24.6 (18.7–31.8)	
South	1,386	38.8 (36.3–41.5)	199	46.6 (40.1–53.3)	
West	758	18.2 (16.6–19.9)	96	15.0 (11.9–18.7)	
**Body mass index, kg/m^2^**					
<25.00	1,144	33.2 (30.7–35.8)	101	25.2 (19.8–31.5)	.10
25.00–29.99	1267	32.1 (29.8–34.5)	133	37.7 (30.5–45.5)	
≥30.00	1,318	34.7 (32.3–37.1)	126	37.0 (29.6–45.2)	
**Current smoking status^e^**					
Yes	543	15.9 (14.2–17.8)	65	22.7 (16.0–31.2)	.055
No	3186	84.1 (82.2–85.8)	269	77.3 (68.8–84.0)	
**Diabetes diagnosis**					
No	3,201	88.3 (87.0–89.4)	405	84.3 (77.8–89.2)	.14
Yes	528	11.7 (10.6–13.0)	64	15.7 (10.8–22.2)	
**Hypertension diagnosis**					
No	2,434	71.8 (69.8–73.6)	300	64.3 (57.6–70.5)	.02
Yes	1,295	28.2 (26.4–30.2)	169	35.7 (29.5–42.4)	

Abbreviation: CI, confidence interval.

^a^ In this study, we linked the HealthStyles and ConsumerStyles data to obtain data on sodium questions of interest. Among 4,198 HealthStyles participants, we excluded 28 (0.7%) respondents who did not have education information and 441 (10.5%) who had incomplete data on all study questions. The final sample was 3,729.

^b^ Unweighted.

^c^ All estimates are weighted for age, sex, race/ethnicity, annual household income, and household size.

^d^ Other race includes Alaska Native, American Indian, Asian, Native Hawaiian, and Other Pacific Islander.

^e^ The possible responses to the question on smoking were the following: “former smoker,” “nonsmoker”, and “smoker.” The categories “nonsmoker” and “former smoker” were combined.
